# Dendritic Cell Based PSMA Immunotherapy for Prostate Cancer Using a CD40-Targeted Adenovirus Vector

**DOI:** 10.1371/journal.pone.0046981

**Published:** 2012-10-08

**Authors:** Briana Jill Williams, Shilpa Bhatia, Lisa K. Adams, Susan Boling, Jennifer L. Carroll, Xiao-Lin Li, Donna L. Rogers, Nikolay Korokhov, Imre Kovesdi, Alexander V. Pereboev, David T. Curiel, J. Michael Mathis

**Affiliations:** 1 Gene Therapy Program, Departments of Urology, Biochemistry and Molecular Biology, and Cellular Biology and Anatomy, and the Feist-Weiller Cancer Center, LSU Health Sciences Center, Shreveport, Louisiana, United States of America; 2 VectorLogics, Inc., Birmingham, Alabama, United States of America; 3 Departments of Medicine and Pathology, University of Alabama at Birmingham, Birmingham, Alabama, United States of America; 4 Department of Radiation Oncology, Washington University School of Medicine, St. Louis, Missouri, United States of America; Carl-Gustav Carus Technical University-Dresden, Germany

## Abstract

Human prostate tumor vaccine and gene therapy trials using *ex vivo* methods to prime dendritic cells (DCs) with prostate specific membrane antigen (PSMA) have been somewhat successful, but to date the lengthy *ex vivo* manipulation of DCs has limited the widespread clinical utility of this approach. Our goal was to improve upon cancer vaccination with tumor antigens by delivering PSMA *via* a CD40-targeted adenovirus vector directly to DCs as an efficient means for activation and antigen presentation to T-cells. To test this approach, we developed a mouse model of prostate cancer by generating clonal derivatives of the mouse RM-1 prostate cancer cell line expressing human PSMA (RM-1-PSMA cells). To maximize antigen presentation in target cells, both MHC class I and TAP protein expression was induced in RM-1 cells by transduction with an Ad vector expressing interferon-gamma (*Ad5-IFNγ*). Administering DCs infected *ex vivo* with CD40-targeted *Ad5-huPSMA*, as well as direct intraperitoneal injection of the vector, resulted in high levels of tumor-specific CTL responses against RM-1-PSMA cells pretreated with *Ad5-IFNγ* as target cells. CD40 targeting significantly improved the therapeutic antitumor efficacy of *Ad5-huPSMA* encoding PSMA when combined with *Ad5-IFNγ* in the RM-1-PSMA model. These results suggest that a CD-targeted adenovirus delivering PSMA may be effective clinically for prostate cancer immunotherapy.

## Introduction

Prostate cancer ranks second among the leading cancer-related deaths in the United States in men, and an estimated 240,890 new cases and 33,720 deaths will have occurred in 2011 [1]. Although treatments are available for organ-confined carcinoma of prostate, there is no effective approach to treat recurrent disease after androgen deprivation therapy fails. This calls for the development of novel strategies to combat this disease. Recent reports suggest suppression of prostate tumor growth is possible following immunization strategies using vaccines encoding tumor antigens [2], [3]. Prostate specific membrane antigen (PSMA) is a type II membrane protein with folate hydrolase activity expressed primarily in prostate epithelium and a limited number of other cell types. This well-defined prostate expression is significantly elevated in prostate cancer, especially in advanced stages [4]. Thus, PSMA is a favorable potential target for prostate cancer immunotherapy. Several PSMA-based vaccines had been developed and clinical trials indicate that these immunotherapy approaches can be safely administered and can induce immune responses in patients with advanced carcinoma of prostate [5], [6]. However, limited clinical responses observed so far warrant an alternative vaccination paradigm.

Dendritic cells (DCs) are the professional APCs that play a potent role in the initiation of immune response by activating T-cells. It is known that interaction of DCs through CD40 with T helper cells expressing the CD40 ligand (CD40L) can aid in their maturation that in turn triggers CTL response. Previous reports have shown that *ex vivo* DC-based vaccines can induce specific anti-tumor T-cell responses in patients [7], [8], [9]. Despite these clinical successes, this approach is limited from widespread clinical application because manipulating DCs through *ex vivo* culture and antigen loading is laborious, expensive, and time consuming. Likewise, *ex vivo* prepared DCs show limited migration to the lymph nodes for subsequent activation of T-cells [10]. This problem has been addressed by *in situ* loading of DCs with tumor associated antigens using viral and non-viral vectors [11]. Among the viral vectors, recombinant adenoviral vectors (Ads) have received much attention for cancer therapy because of their high capacity and robust gene expression [12]. Nonetheless, Ad vectors poorly infect DCs because of a lack in expression of the Coxsackie and adenovirus receptor mediating infectious uptake [13]. This limitation could be overcome by using a bispecific adapter molecule that encompasses a fusion of an extracellular domain of the native Coxsackie and adenovirus receptor receptor and the mouse CD40 ligand linked by a trimerization motif from the T4 bacteriophage fibritin protein 14,15. More recently, this adapter was used successfully for DC-based immunotherapy in a mouse model of melanoma [16], [17]. However, other tumor/antigen combinations have not been tested.

In the present study, we evaluated a dendritic cell-targeted Ad vaccine expressing human PSMA *in vivo* in a mouse model of prostate cancer. We generated an immunocompetent model using the RM-1 mouse prostate cancer cell line that form tumors in syngeneic C57BL6 mice [18], by constitutively expressing the human PSMA antigen. Herein, we show that *in vivo* delivery of a CD40-targeted Ad5 vector leads to increased cytotoxic T cell responsiveness and enhanced therapeutic efficacy in this model. We also demonstrate that IFNγ as an immunological adjuvant in our vaccine regime increased antigen presentation in target cells and maximized this effect.

**Figure 1:**
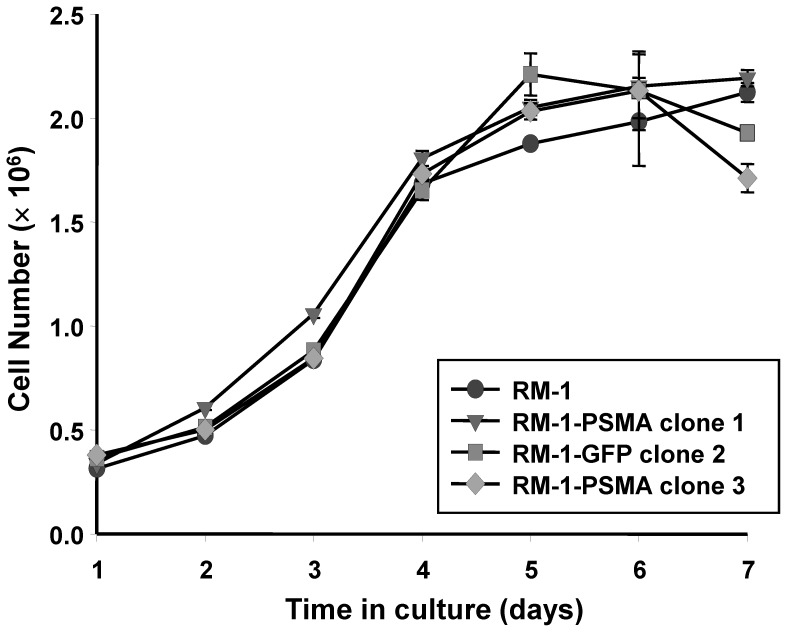
Growth characteristics of transfected cell lines *in vitro*. Shown is a representative growth curve of the number of RM-1 cells (•) in culture with time compared with the number of RM-1-PSMA clone 1 cells (▾), RM-1-PSMA clone 3 cells (⧫), and RM-1-GFP clone 2 cells (▪). Each data point represents the mean ± standard error of three independent experiments.

## Materials and Methods

### Ethics Statement

All animals used in this study received humane care based on guidelines set by the American Veterinary Association as well as in accordance with the *Guide for the Care and Use of Laboratory Animals* (Institute for Laboratory Animal Research, Washington, DC). The experimental protocols involving live animals were reviewed and approved by the Institutional Animal Care and Use Committee of LSU Health Sciences Center at Shreveport (protocol P-10-040). All efforts were made to minimize animal suffering, to reduce the number of animals used and to utilize alternatives to *in vivo* techniques, if available.

**Figure 2:**
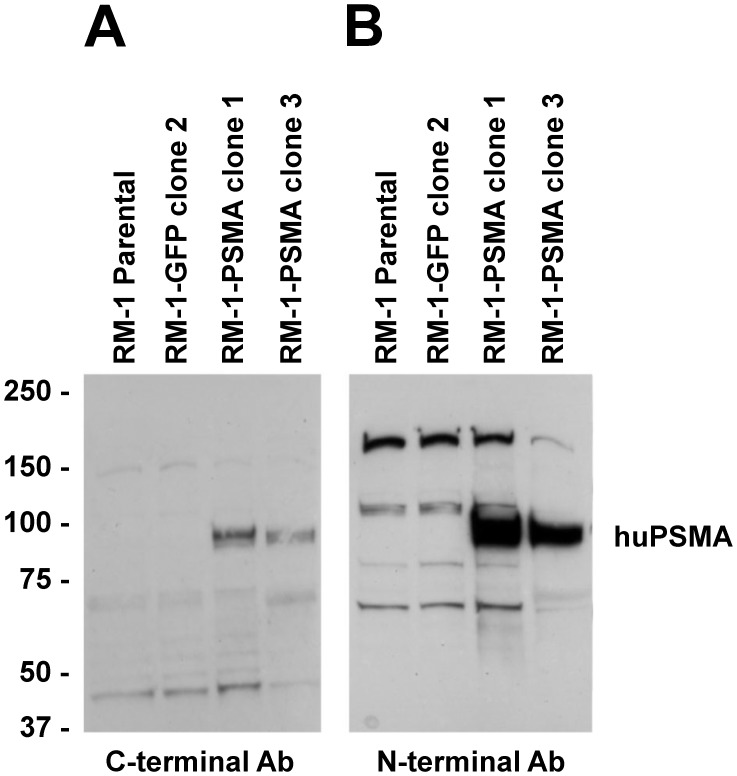
Western blot analysis of PSMA protein levels in RM-1 mouse prostate cancer cell lines. Protein extracts (20 µg) were prepared from each cell line and diluted with RIPA sample buffer. The extracts were separated by SDS-PAGE, electroblotted onto nitrocellulose and probed with an anti-human PSMA antibody directed to the C-terminal protein sequence (A) or an anti-human PSMA antibody directed to the N-terminal protein sequence (B), followed by treatment with corresponding anti-species IgG HRP conjugates. Shown are representative blots after visualization by autoradiography.

### Cell Lines

RM-1, an androgen-insensitive MHC class I–deficient mouse prostate cancer cell line, which is syngeneic in C57BL/6 mice [18], was obtained from Dr. Timothy C. Thompson (Baylor College of Medicine, Scott Department of Urology, Houston, TX). The human transformed embryonic kidney HEK-293 cell line was obtained from the American Type Culture Collection (ATCC; Manassas, VA). The cells were maintained at 37°C and a 5% humidified CO_2_ atmosphere in Dulbecco’s Minimum Essential Medium (DMEM, Mediatech Inc.; Manassas, VA), containing 10% fetal bovine serum (FBS, Gemini Bioproducts; Woodland, CA) and 1% antibiotic-antimycotic solution (Mediatech Inc.; Manassas, VA).

**Figure 3:**
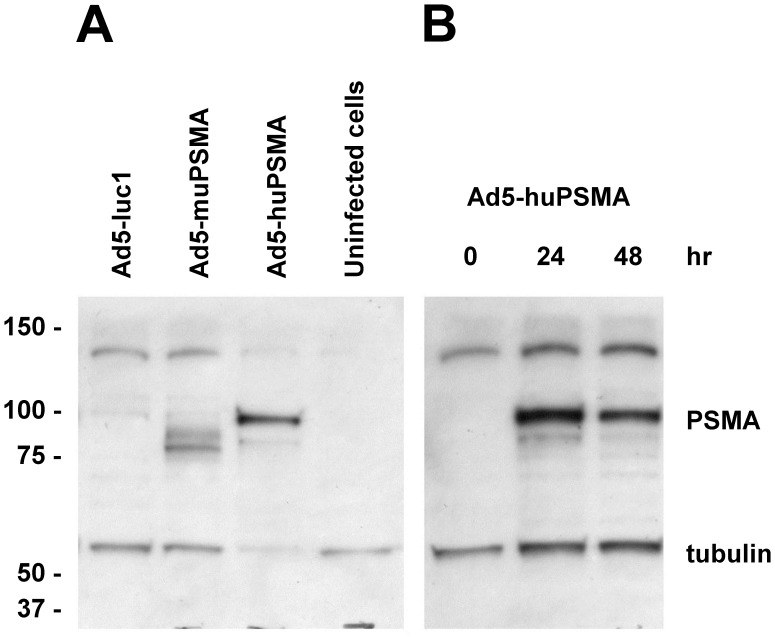
Western blot analysis of PSMA protein levels in adenovirus-infected dendritic cells. (A) Protein extracts (20 µg) were prepared from cultured mouse dendritic cells after infection for 24 h with the CD40-targeted *Ad5-luc1* or CD40-targeted *Ad5-mPSMA* expressing mouse PSMA or *Ad5-huPSMA* expressing human PSMA. (B) Protein extracts (20 µg) were prepared from cultured mouse dendritic cells after infection for 0, 24, or 48 h with the CD40- *Ad5-huPSMA* expressing human PSMA. Each cell extract was prepared using RIPA sample buffer. The extracts were separated by SDS-PAGE, electroblotted onto nitrocellulose membranes and probed with an anti-human PSMA antibody followed by treatment with the corresponding anti-mouse IgG HRP conjugates. Shown are representative blots after visualization by autoradiography. A loading control was used by co-treatment of the membranes with an anti-mouse α-tubulin antibody.

### Animals

Male C57BL/6 mice at 4–6 weeks of age were obtained from Charles River Laboratories (Wilmington, MA).

**Figure 4:**
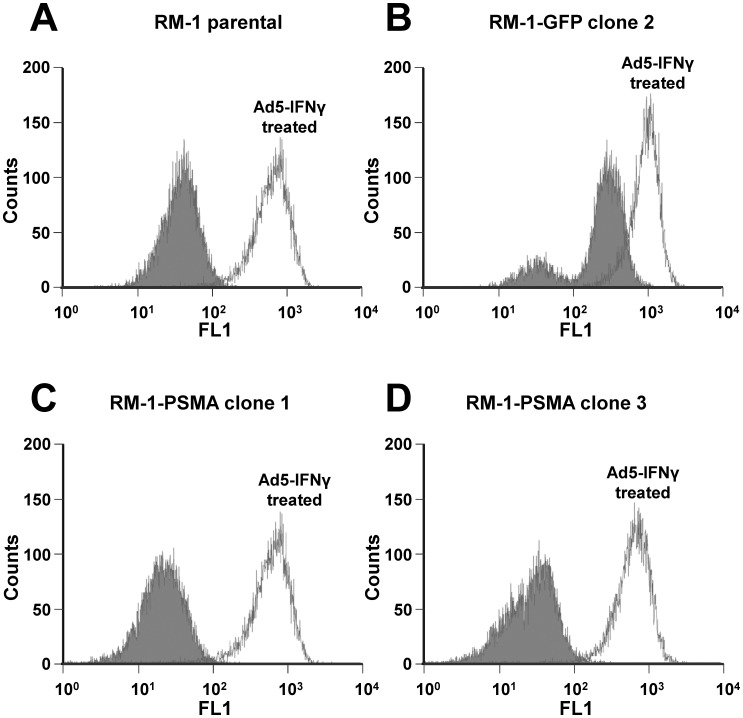
Flow cytometry analysis of MHC class I cell surface expression in RM-1 cells maintained in the absence and presence of IFNγ. Evaluation of binding of anti-MHC class I (H-2Db and H-2Kb) antibodies to (A) RM-1 parental cells, (B) RM-1-GFP clone 2 cells, (C) RM-1-PSMA clone 1 cells, and (D) RM-1-PSMA clone 3 cells. Shown are histogram peaks corresponding to untreated (shaded) and cells infected with *Ad5-IFNγ* (unshaded).

### Generating RM-1 Tumor Cells Expressing Recombinant Human PSMA and GFP

Human elongation factor 1α-subunit promoter (EF-1α) was chosen as a transgene promoter. The human PSMA and the GFP coding sequences were cloned into pEF1/Myc-His vector (Invitrogen; Carlsbad, CA). Constructs with the correct restriction pattern were selected and sequenced, and were used for transfection of RM-1 cells. Cells were then selected by 0.8 mg/mL G418 (Alexis) for 2 weeks to obtain single clone. Expression of recombinant human PSMA expression in antibiotic resistant clones was determined by Western blot analysis (using the method described below) with an anti-PSMA monoclonal antibodies recognizing the N-terminus (7E11C5; previously purified from a hybridoma obtained at ATCC) or the C-terminus (YPSMA-1, Abcam; Cambridge, MA). Two clones expressed detectable level of PSMA (RM-1-PSMA clone 1 and RM-1-PSMA clone 3). They were propagated and frozen for further analysis. The RM-1 clones expressing GFP were identified by fluorescence microscopy. One positive clone (RM-1-GFP clone 2) was expanded and frozen for further analysis.

**Figure 5:**
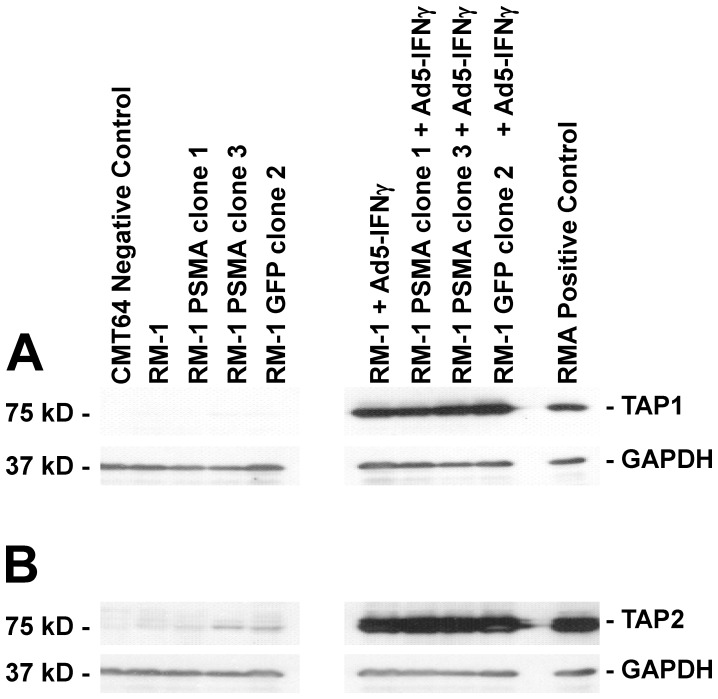
Analysis of TAP expression in RM-1 cell lines induced by *Ad5-IFNγ.* Protein extracts (20 µg) were prepared from each cell line and diluted with RIPA sample buffer. The extracts were separated by SDS-PAGE, electroblotted onto nitrocellulose and probed with (A) an anti-mouse TAP1 antibody or (B) an anti-mouse TAP2 antibody, followed by treatment with a corresponding anti-mouse IgG HRP conjugates. Shown are representative blots after visualization by autoradiography. A loading control was used by co-treatment of the membranes with an anti-mouse GAPDH antibody.

**Figure 6:**
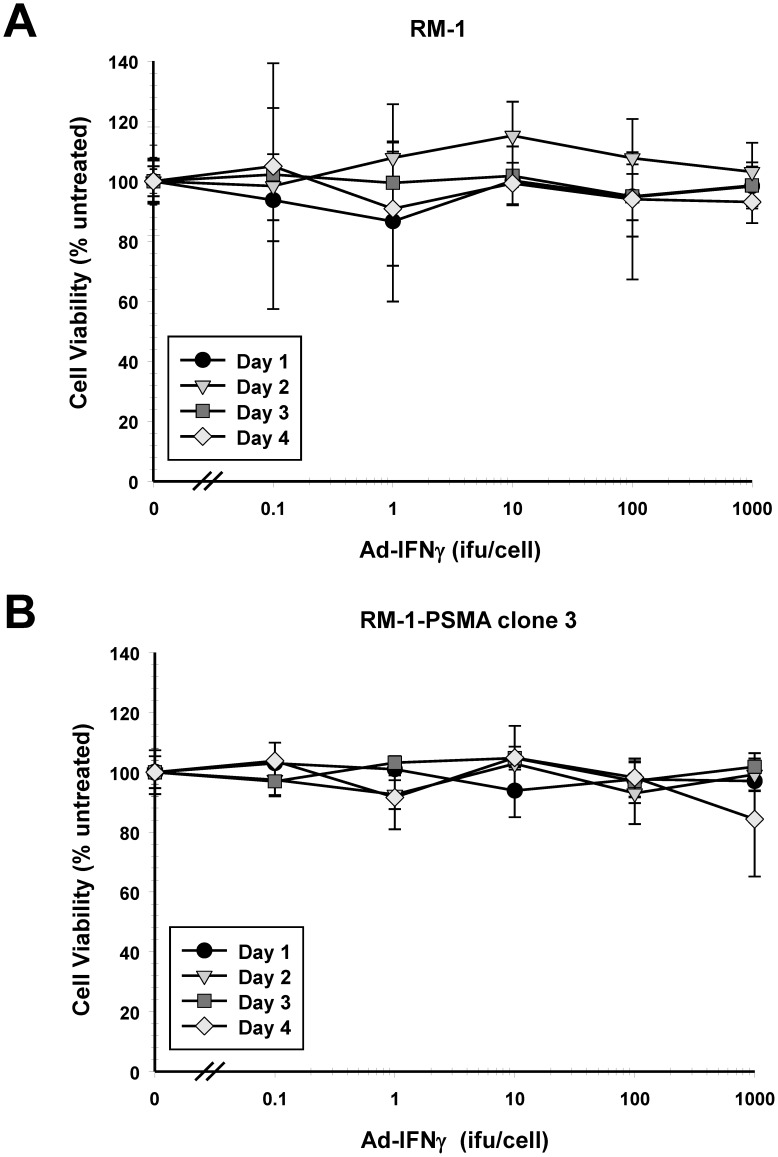
Effect of cell viability on cell lines after infection *in vitro* with *Ad5-IFNγ*. Shown is a representative curve of the cell viability in (A) RM-1 cells or (B) RM-1-PSMA clone 3 cells infected in culture with increasing concentrations of *Ad5-IFNγ* compared with uninfected cells. Effect of cell viability was determined at day 1 (•), day 2 (▾), day 3 (⧫), and day 4 (▪) after initiation of infection with *Ad5-IFNγ*. Each data point represents the mean ± standard error of three independent experiments.

### Adenoviral Vectors

*Ad5-huPSMA* was constructed by subcloning the human PSMA cDNA coding sequence into the MfeI - BstXI sites of the pAdenoVatorCMV5 shuttle vector (QBiogene; Carlsbad, CA). The resulting pAdenoVatorCMV5-hu-PSMA shuttle vector was used to construct an adenovirus by homologous recombination with pAdEasy1 (containing the E1 and E3 deleted Ad5 backbone) in *E. coli* using methods previously described [19]. The resultant recombinant plasmid was linearized with Pac I and transfected into HEK-293 cells to generate the *Ad5-huPSMA* virus. The *Ad5-luc1*, a human Ad serotype 5 vector containing a firefly luciferase expression cassette within the E1 deleted region was provided by Dr Igor Dmitriev (Washington University School of Medicine, St. Louis, MO). The *Ad5-IFNγ*, a human Ad serotype 5 vector, consisting of an E1-deleted human serotype 5 Ad with a chicken β-actin promoter driving the expression of the mouse IFNγ cDNA was provided by Dr. Hirofumi Hamada (Department of Molecular Medicine, Sapporo Medical University, Sapporo, Japan). The adenovirus stocks were propagated and amplified using the human transformed embryonic kidney HEK-293 cell line. High titer adenovirus stocks were purified using an Adenopure purification kit (Puresyn, Inc, Malvern, PA). Following purification the viruses were quantified using an Adeno-X Rapid Titer Kit (BD Biosciences, Palo Alto, CA). The viruses were dialyzed overnight in dialysis buffer (potassium phosphate buffered saline containing 1 M sucrose and 0.5% β-cyclodextrin) at 4°C before storage at −80°C.

**Figure 7:**
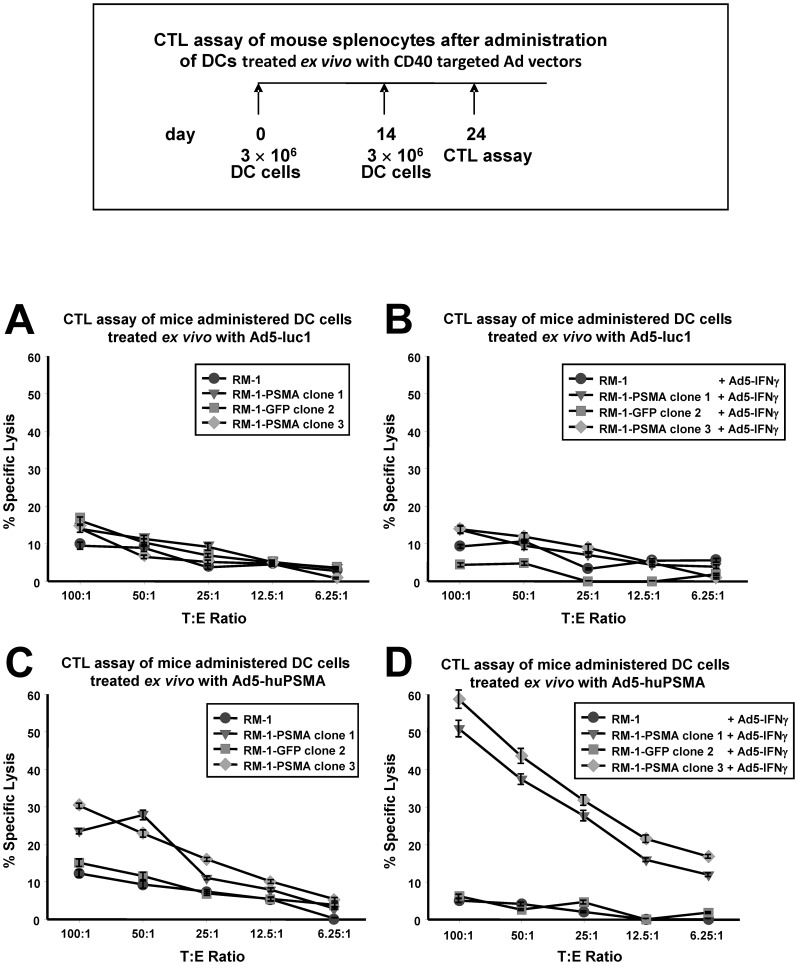
CTL assays of T-cells from mice after *ex vivo* infection of DCs with the CD40-targeted Ad5 vectors expressing PSMA or luciferase followed by intraperitoneal administration. Mice were immunized as described in Material and Methods with dendritic cells infected with the CD40-targeted *Ad5-luc1* or with the CD40-targeted *Ad5-huPSMA*. At day 24 after initiation of the experiment, the animals were euthanized, and CTL activity was measured in cells harvested from the spleens. Target cells used were RM-1 parental (•), RM-1-PSMA clone 1 cells (▾), RM-1-PSMA clone 3 cells (⧫), and RM-1-GFP clone 2 cells (▪). Target cells were either untreated (A and C) or pre-treated by infection with *Ad5-IFNγ* (B and D). Each data point represents the mean ± standard error of four replicate wells.

### CD40 Targeting Ligand

A recombinant molecular adaptor protein consisting of the soluble extracellular domain of the Coxsackie and adenovirus receptor linked to the mouse CD40 ligand via a trimerzation motif (CFm40L) was constructed, produced and purified as described previously [14], and used to retarget adenoviral vectors to mouse DC.

**Figure 8:**
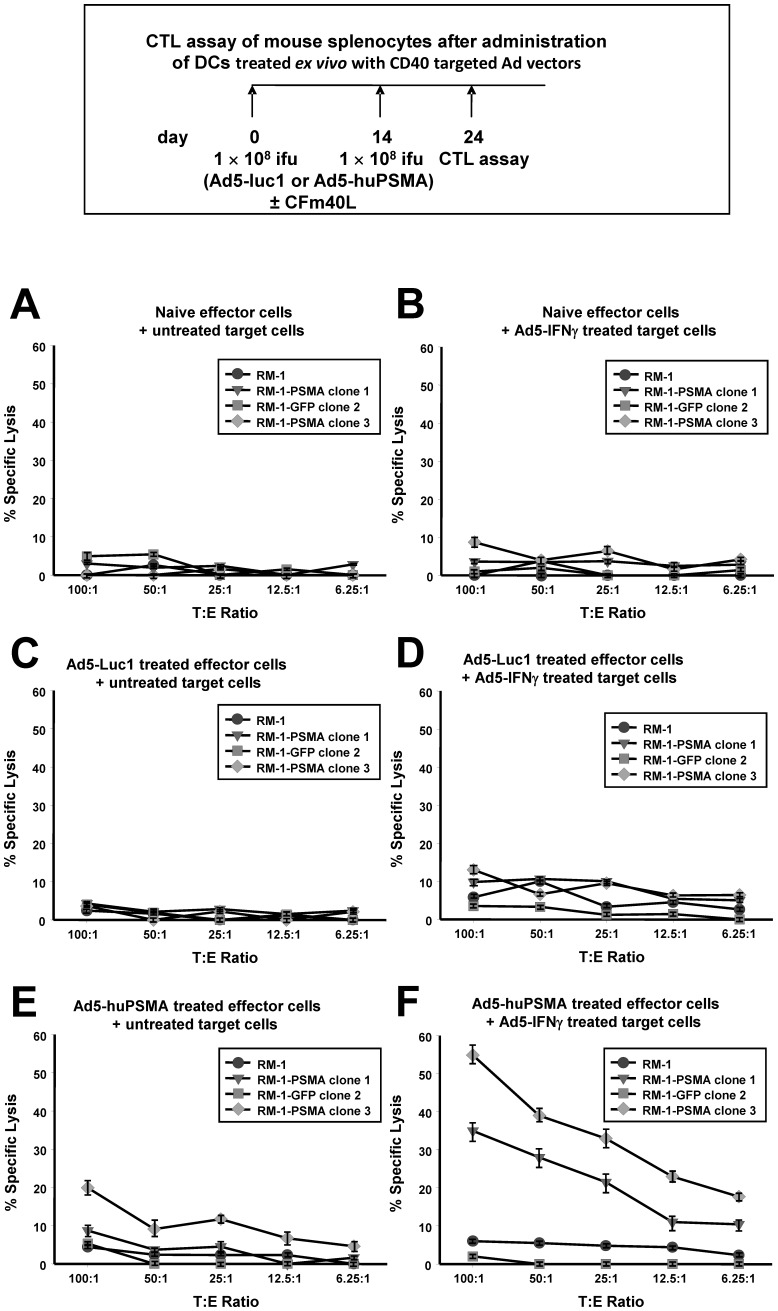
CTL assays of T-cells from mice after direct intraperitoneal administration of CD40-targeted vectors expressing PSMA or luciferase. Mice were immunized as described in Material and Methods with the CD40-targeted *Ad5-luc1* or with the CD40-targeted *Ad5-huPSMA*. At day 24 after initiation of the experiment, the animals were euthanized, and CTL activity was measured in cells harvested from the spleens. As a control, CTL activity was measured in cells harvested from spleens of naïve (untreated) mice. Target cells used were RM-1 parental (•), RM-1-PSMA clone 1 cells (▾), RM-1-PSMA clone 3 cells (⧫), and RM-1-GFP clone 2 cells (▪). Target cells were either untreated (A, C, and E) or pre-treated by infection with *Ad5-IFNγ* (B, D, and F). Each data point represents the mean ± standard error of four replicate wells.

### Western Blot Analysis of TAP Expression

Control RM-1 cells and RM-1 cells incubated for 24 h with *Ad5-IFNγ* at multiplicity of infection (MOI) of 100 ifu/cell were harvested and lysates were prepared in RIPA sample buffer. Protein concentrations of the samples were normalized by Bradford assay. Samples were run on 10% sodium dodecyl sulfate-polyacrylamide gel electrophoresis (SDS-PAGE) gels and transferred to nitrocellulose membranes. Membranes were blocked for 1 h with 5% bovine serum albumin (BSA) and washed with TTBS (1% Tween-20 in Tris-buffered saline). Afterwards, the membranes were incubated with anti-mouse TAP1, anti-mouse TAP2, or anti-mouse GAPDH antibodies (Santa Cruz Biotechnology; Santa Cruz, CA) for 1 h. After three consecutive washes with TTBS, membranes were incubated with horseradish peroxidase-conjugated goat anti-mouse IgG antibody for 1 h, and washed three times for 30 min each with TTBS. Finally the membranes were developed using the ECL substrate (Amersham; Piscataway, NJ), and exposed to X-ray film.

**Figure 9:**
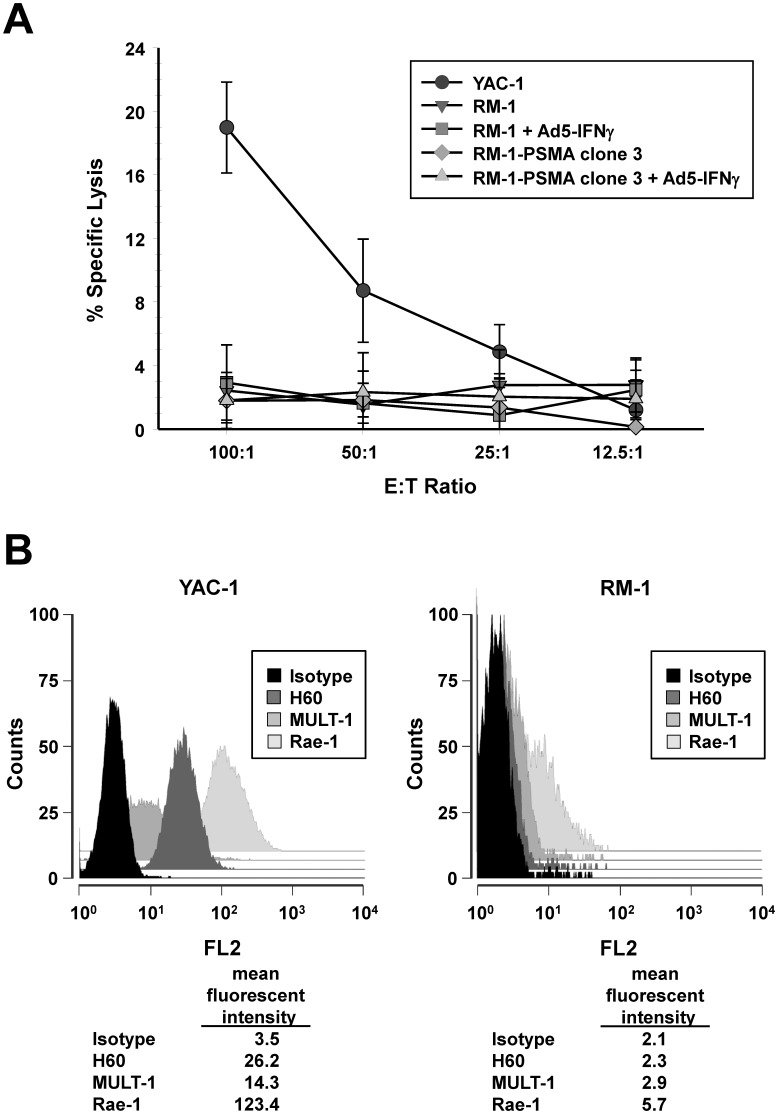
Assessment of sensitivity to NK cell-mediated cytotoxicity. (A) Cytotoxic activity of NK effector cells on YAC-1 and RM-1 target cells was determined by a ^51^Cr-release assay at indicated E:T ratios. Target cells used were YAC-1 (•), RM-1 parental (▾), RM-1 parental cells pre-treated by infection with *Ad5-IFNγ* (▪), RM-1-PSMA clone 3 cells (⧫), or RM-1-PSMA clone 3 cells pre-treated by infection with *Ad5-IFNγ* (▴). Each data point represents the mean ± standard error of four replicate wells. (B) Flow cytometry analysis of YAC-1 and RM-1 cells stained with PE labeled anti-H60, anti-MULT-1, or anti-Rae-1 antibodies or with an isotype-matched control antibody. Numbers below the histograms correspond to mean fluorescence intensity of each peak.

**Figure 10:**
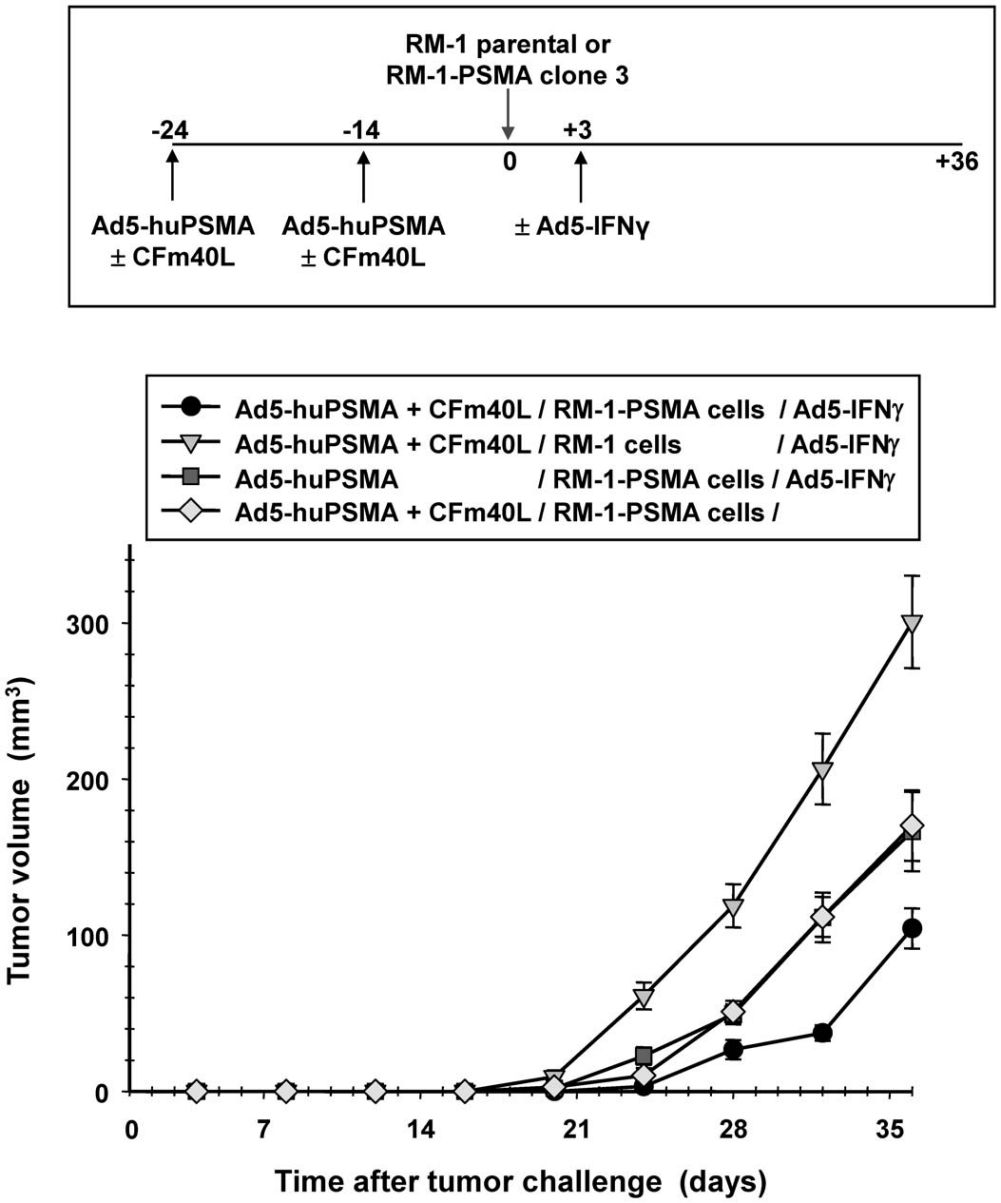
Assessment of tumor growth after vaccine treatment. Antitumor effect of a CD40-targeted *Ad5-huPSMA* vaccine was determined using the RM-1-PSMA mouse model. Mice were immunized as described in Material and Methods with the CD40-targeted *Ad5-luc1* or CD40-targeted *Ad5-huPSMA*. At day 24 after initiation of the experiment, each mouse received 4×10^6^ RM-1 parental cells or 4×10^6^ RM-1-PSMA clone 1 cells injected subcutaneously. Three days later, the treatment groups were injected at the site of tumor cell injection with 1×10^8^ ifu of *Ad5-IFNγ* or with normal saline. Beginning at the time of tumor cell challenge (day 0), the tumors were measured and volumes calculated by the formula: *tumor volume = *½ × (*length*×*width* ^2^) where length is the longest distance of the tumor. Each data point represents the mean volume of 15 tumors ± standard error.

### Flow Cytometry Analysis of MHC Class I Expression

RM-1 cells (parental as well as RM-1-PSMA clones expressing human PSMA) were analyzed for the cell surface expression of MHC class I (Db and Kb). Briefly, RM-1 cells were infected for 24 h with *Ad5-IFNγ* at MOI 100. At 24 h post-incubation, cells were harvested and immunostained by using a fluorescein isothiocyanate (FITC) labeled anti-mouse H-2Kb/H-2Db antibody (BD Biosciences; San Jose, CA). This antibody reacts with the mouse H-2Kb MHC class I alloantigen haplotype and cross-reacts with H-2Db, but does not react with other haplotypes. Cells were incubated with the antibody (1∶1000 dilution) for 20 min at 4°C and washed 3 times in ice-cold PBS. An isotype antibody (mouse IgG2a, κ) was also used to immunostain cells as a negative control. Subsequently, the cells were fixed in 1% paraformaldehyde for 5 min in the dark, washed twice with ice-cold PBS and resuspended in 200 uL of PBS buffer to be analyzed by flow cytometry (FACSCalibur; BD Biosciences).

### Preparing Bone Marrow-derived DCs

Bone marrow-derived DCs were generated from 4 to 6 weeks old C57BL/6 male mice. DCs were cultured as described previously [20] with some modification. DCs were cultured in RPMI-1640 medium containing 10% FBS (Atlanta Biosciences), with 10 ng/mL each of recombinant granulocyte–macrophage colony-stimulating factor (GM-CSF) and Interleukin-4 (IL-4) from PeproTech Inc. (Rock Hill, NJ). On day 3 of culture, fresh medium was added to the DC cultures. On day 6 of culture, DCs were exposed for 4 hr to the CD40-retargeted *Ad5-huPSMA* at MOI 100. Subsequently, the DCs were matured by incubating with 100 ng/mL lipopolysaccharide (LPS, Sigma; St Louis, MO) overnight. The matured DCs were washed twice with PBS and administered by intraperitoneal (i.p.) injection into C57BL/6 mice.

### Immune Response after Vaccination using DCs Stimulated *ex vivo* with Ad5-huPSMA

The mice were vaccinated with Ad-infected DCs (1×10^6^ cells/mouse) suspended in sterile PBS in a total volume of 50 µL by i.p. injection on day 0 and 14. On day 24, the mice were euthanized and spleens were harvested. Single cell suspensions were prepared by mincing the spleens in serum-free RPMI medium with scissors and gentle repeated pipetting, followed by passing the suspensions through 100 µM nylon mesh. The red blood cells were lysed with lysis buffer, and the purified T-cells were washed twice in serum-free medium, counted, and seeded at a density of 1×10^6^ cells per well of 24-well plates in 500 µL of serum-free medium.

### Immune Response after Direct Vaccination *in vivo* with Ad5-huPSMA

C57BL/6 mice were immunized by direct i.p. injections with *Ad5-huPSMA*. Each mouse was injected with 1×10^9^ ifu of *Ad5-huPSMA* alone or 1×10^9^ ifu of *Ad5-huPSMA* complexed with 1200 ng of CFm40L on day 0. A second dose was administered 14 days later. At 24 days after the initial immunization, the mice were euthanized and the spleens were collected and T-cells cultured as described above.

### Generating CTLs and Performing Cytotoxicity Tests

Cultured T-cells from the mouse spleens were re-stimulated for 6 days by the addition of irradiated HEK-293 cells infected with *Ad5-huPSMA* to over express the PSMA antigen. Following re-stimulation, T-cells were harvested and separated from the dead cells. The cytotoxicity was determined in a standard 4 h chromium release assay using the stimulated T-cells as effector cells (E) and RM-1 cell lines as specific targets (T) at the indicated E:T ratios. The RM-1 cell lines (RM-1-PSMA clone 1, RM-1-PSMA clone 3, and RM-1-GFP clone 2) were used directly as target cells or were previously incubated for 24 h with *Ad5-IFNγ* (MOI 100). The cells were labeled with 100 µCi of Na_2_^51^CrO_4_ for 1.5 h at 37°C. Subsequently, 1×10^3^ labeled target cells were co-cultured with different numbers of effector cells in a 96-well (v-bottom plates) for 4 h at 37°C. At the end of the culturing, 100 µL of supernatants were collected and radioactivity was measured on a gamma-counter. Maximum and spontaneous release of ^51^Cr was obtained from the supernatants of the target cells in 1% Nonidet P-40 and in medium alone respectively. All experiments were performed with quadruplicate wells. The ^51^Cr-release was measured by a gamma counter (LKB Instruments, Gaithersburg, MD), and the percent specific lysis was calculated by the following formula: *% specific lysis = [(experimental c.p.m. - spontaneous c.p.m.)/(maximal c.p.m. - spontaneous c.p.m.)]* ***×*** *100.*

### Flow Cytometry Analysis of NKG2D Ligand Expression

RM-1-PSMA clone 3 cells and YAC-1 cells were plated at 100,000 cells/well into 24-well tissue culture dishes. The cells were allowed to attach by overnight incubation at 37°C. On the following day, the cells were incubated in the absence or presence of *Ad5-IFNγ* at 100∶1 MOI at 37°C in serum-free media. At 2 h post-infection, the virus-containing media was replaced with complete growth medium containing 10% FBS. After 48 h, the untreated or *Ad5-IFNγ* treated cells were harvested and washed twice with PBS. The cells were incubated for 1 h at 4°C with phycoerythrin (PE) labeled rat IgG_2A_ isotype control antibody (R&D Systems, Minneapolis, MN) or with the following antibodies: (a) a PE labeled rat anti-mouse H60 monoclonal antibody (R&D Systems), (b) a PE labeled rat anti-mouse Rae-1 (pan-specific) monoclonal antibody (R&D Systems), or (c) a PE labeled rat anti-mouse MULT-1 monoclonal antibody (R&D Systems). Following the antibody incubation, the cells were washed twice with PBS, resuspended in 0.4 ml PBS, and analyzed by flow cytometry using a FACS Calibur instrument (Becton Dickinson, San Jose, CA).

### Cell Viability Assay

RM-1 and RM-1-PSMA clone 3 cells were seeded onto 96-well plates (2,000 cells/well) with DMEM containing 10% fetal bovine serum. Following an overnight incubation, the cells were infected with *Ad5-IFNγ* at increasing MOI (0, 0.1, 1, 10, 100, 1000 ifu/cell) in serum-free medium at 37°C. The *Ad5-IFNγ* containing medium was aspirated after 2 h and replaced with complete growth medium. A cell viability assay was performed at days 1, 2, 3, and 4 post-infection using the CellTiter-Blue reagent (Promega, Madison, WI) by following manufacturer’s instructions. Briefly, at each time point, 20 µL of CellTiter-Blue reagent was added to the wells and mixed for 10 sec on a shaker. The cells were then incubated for another 22 h at 37°C. Afterwards, the fluorescence signal in each well was analyzed using a Fluoroskan Ascent microplate fluorometer (Thermo Fisher Scientific, Waltham MA) with an excitation setting at 530 nm and an emission setting at 620 nm.

### Splenic Effector Cell Isolation

Control (untreated) male C57/BL6 mice were euthanized by CO_2_ asphyxiation. Spleens were harvested and homogenized manually between the ends of frosted glass microscope slides and passed through a 70-µ mesh. Dispersed splenic cells were centrifuged at 1,000 rpm in a tabletop centrifuge for 5 min and resuspended in red blood cell (RBC) lysis buffer (138 mM NH_4_Cl, 1 mM KHCO_3_, and 0.1 mM EDTA) for 5 min. The remaining splenocytes were washed three times in ice-cold PBS. Cell suspensions were counted and adjusted to 1×10^7^ cells/mL.

### Chromium Release Assay

To measure sensitivity of RM-1 cells to mouse NK cell lysis, a chromium release assay was performed. Initially, RM-1 cells or RM-1-PSMA clone 3 cells were plated into 100 mm tissue culture dishes. The cells were allowed to attach by overnight incubation at 37°C. On the following day, the cells were incubated in the absence or presence of *Ad5-IFNγ* at 100∶1 MOI at 37°C in serum-free media. At 2 h post-infection, the virus-containing media was replaced with complete growth medium containing 10% FBS. After 48 h, the untreated or *Ad5-IFNγ* treated cells were harvested by trypsinization and labeled with ^51^Cr. The target cells were counted and resuspended at a concentration of 1×10^7^ cells/mL of medium. To each cell suspension, 125 µCi of ^51^Cr (ICN Pharmaceuticals, Costa Mesa, CA) was added as an aqueous solution of sodium chromate and incubated for 90 minutes at 37°C with swirling every 15 minutes. The labeled target cells were washed once in PBS and three times in complete medium. To initiate the chromium release assay, the isolated splenocyte effector cells were added to the wells of U-bottomed 96-well plates (100, 50, 25, and 12.5 µL for each cell target) in replicates of four. All wells were brought to a final volume of 100 µL with complete medium. Two sets of controls were employed instead of effector cells: one set contained only 100 µL of complete medium to account for spontaneous ^51^Cr release and one set contained 100 µL of 2N HCl to account for maximum ^51^Cr release. The labeled target cells were adjusted to 1×10^5^ cells/mL, and 100 µL was quickly added to each of the wells on the plate. The plates were then incubated at 37°C for 4 hours. Following incubation, the plates were centrifuged at 1,200 rpm for 5 minutes to pellet the cells. A portion (100 µL) of the resulting supernatant was removed to glass tubes for analysis using a gamma counter, and the percent specific lysis was calculated by the following formula: *% specific lysis = [(experimental c.p.m. - spontaneous c.p.m.)/(maximal c.p.m. - spontaneous c.p.m.)] ×100.*

### Tumor Challenge Experiment

Groups of fifteen C57BL/6 mice were immunized by direct i.p. injection with *Ad5-huPSMA*. Each mouse was injected with 1×10^9^ ifu of *Ad5-huPSMA* alone or 1×10^9^ ifu of *Ad5-huPSMA* complexed with 1,200 ng of CFm40L on day 0. A second dose was administered 14 days later. The parental RM-1 cell line or the RM-1-PSMA clone 1 cell line were harvested from cell culture flasks using 1% trypsin and washed with PBS. Subsequently, the cells were injected into the C57BL/6 mice immunized as described above. Each mouse received 4×10^5^ cells injected at day 24 after initiation of the immunization regimen. Subsequently, tumor volume was measured with a digital caliper (VWR INTERNATIONAL; WAYNE, PA) every four days. At each tumor measurement, the greatest longitudinal diameters (length) and the greatest transverse diameters (width) were measured, and the tumor volumes were estimated using the formula: *tumor volume = *½ × (*length* × *width* ^2^).

### Statistical Analysis

Data are presented as mean ± standard error (SEM) of the data points. Statistical analysis was performed using Student’s t-test, using GraphPad Prism 5.0 software (GraphPad Software, Inc.; La Jolla, CA). Data were considered statistically significant when P<0.05.

## Results

### RM-1 Mouse Prostate Cancer Cell Lines Expressing Human PSMA

The full-length human PSMA cDNA sequence was cloned into mammalian expression plasmid vector pEF1/V5-His downstream of the enhancer/promoter elements from the human elongation factor 1α subunit (hEF-1α) for high-level expression in mammalian cells. As a negative control, the green fluorescent protein (GFP) cDNA was also cloned into the pEF1/V5-His expression vector. The mouse prostate cancer cell line RM-1 was transfected with the expression vectors, and individual clones were isolated by G418 selection followed by limiting dilution. Three individual clones were isolated: RM-1-PSMA clone 1 and RM-1-PSMA clone 3 express the human PSMA cDNA and RM-1-GFP clone 2 expresses the GFP cDNA. In order to characterize the growth properties of the clones in culture, the cell numbers were measured over 7 days of culturing. As shown in *Fig. 1*, the results demonstrate that under normal growth conditions, all three RM-1 cell lines grow in a similar fashion as the parental RM-1 cell line.

### Western Blot Analysis of PSMA Expression

To determine the level of PSMA expression in the selected RM-1 clones, Western blot analysis was performed using rabbit polyclonal antibodies prepared from peptides corresponding to the C-terminal sequence or the N-terminal sequence of the human PSMA. Lysates from the two RM-1-PSMA clones were loaded on gel and compared to the lysates from control (parental) RM-1 cells and RM-1-GFP cells. As shown in *Fig. 2A*, predicted bands of approximately 100 kDa was visualized in the RM-1-PSMA clone 1 and clone 3 but not in the parental RM-1 cells or in RM-1-GFP cells. Levels of PSMA expression were similar in both RM-1-PSMA clones. In addition, PSMA detection with antibodies targeting either N-terminal or C-terminal sequences revealed identical staining pattern with no sign of abnormal expression or protein degradation (*Fig. 2B*).

### Constructing a CD40-targeted Ad Vector to Deliver PSMA to DCs

Previous studies have demonstrated that targeting to mouse CD40 enhanced Ad-mediated gene transfer to mouse DC, which was accompanied by phenotypic changes characteristic of DC maturation [14]. This targeting approach was achieved using an adapter molecule that bridges the fiber of the Ad5 to CD40 on mouse DC. We utilized an Ad vector, which incorporated a human PSMA expression cassette within the E1A region under the control of the CMV promoter (*Ad5-huPSMA*). We then tested this vector on the ability to infect mouse DCs, which has been shown previously to poorly express the Coxsackie and adenovirus receptor but to be CD40 positive [21]. The optimal CFm40L concentration (lowest amount of CFm40L achieving maximal DC infection) was determined to be 120 ng CFm40L per 1×10^8^ ifu (infectious units) of virus at a multiplicity of infection (MOI) at 100 ifu *Ad5-huPSMA* per cell (data not shown). Western blot analyses confirmed PSMA expression (*Fig. 3A*) in cells infected with *Ad5-huPSMA* which peaked at 24 h post-infection and was maintained for up to 48 h (*Fig. 3B*). These experiments demonstrate efficient infection of mouse DCs with *Ad5-huPSMA* and efficient expression of the PSMA antigen.

### Cell Surface Expression of MHC Class IA Molecules in Response to IFNγ

RM-1 cells have been previously shown to express low levels of MHC class I (Db and Kb) [22], which can be up regulated by treatment with recombinant IFNγ [23]. Cell surface expression of MHC class I molecules on RM-1 cells after infection with *Ad5-IFNγ* was determined by flow cytometry. Low basal cell surface expression of MHC class I antigens was observed for the parental RM-1 cell line as well as the RM-1-PSMA clones 1 and 3 and the RM-1-GFP clone 2 cell lines in the absence of *Ad5-IFNγ* treatment. Treatment with 100 ifu *Ad5-IFNγ* per cell for 24 h dramatically increased MHC class I cell surface expression on each of the RM-1 cell lines compared with untreated control cells. The results of this experiment show that the RM-1 and all of the clonal cell lines have low cell surface expression of MHC class I, while treatment of the cells with *Ad5-IFNγ* showed marked up regulation of MHC class I expression *(* *Figs. 4A - D* *)*.

### Western Blot Analysis of TAP1 and TAP2 Protein Expression in RM-1 Cell Lines

Antigenic peptides are generated in the proteasome of target cells, and then translocated across the membrane of the endoplasmic reticulum by the transporter associated with antigen processing (TAP). TAP is a trimeric complex consisting of TAP1, TAP2 and tapasin [24]. To characterize the antigen presentation pathway in RM-1 cells, expression of TAP1 and TAP2 proteins was analyzed by Western blotting. As shown in *Fig. 5A* and *Fig. 5C*, TAP1 protein levels were undetectable and TAP2 protein levels were barely detectable in any of the RM-1 cell lines. However, treatment with *Ad5-IFNγ* for 24 h dramatically increased both TAP1 and TAP2 protein expression in each of the RM-1 cell lines *(* *Fig. 5B* *and* *Fig. 5D* *)* compared with the untreated cells. These results demonstrate that *Ad5-IFNγ* induces expression of the MHC class I antigen presentation pathway in RM-1 cells.

### Effect of *Ad5-IFNγ* on Cellular Viability

IFNγ can activate and suppress a number of different target genes, leading to decreased cellular viability by inducing cell cycle arrest and apoptosis. To determine any effect IFNγ had on cellular viability, RM-1 and RM-1-PSMA clone 3 cells were infected with *Ad5-IFNγ* ranging from 0.1 to 1,000 ifu/cell and assayed at days 1, 2, 3, and 4 after infection. As shown in *Fig. 6* *,* cell viability was unchanged in cells infected at any concentration of *Ad5-IFNγ* compared with untreated cells. No decrease was observed up to 4 days after infection, either in RM-1 cells (*Fig. 6A*) or in RM-1-PSMA clone 3 cells (*Fig. 6B*); these results indicate that there is no direct effect of IFNγ on cellular viability *in vito*.

### CTL Assay of Mouse Splenocytes after *in vivo* Administration of DCs Treated *ex vivo* with the CD40-targeted Ad Vectors

To study the effect of CD40-targeted Ad infection on DC-mediated CTL activation, we used two CD40-targeted Ad vectors: *Ad5-luc1* or *Ad5-huPSMA*. In the experiment shown in *Fig. 7*, C57BL/6 mice were immunized with 3×10^6^ DCs infected with either the CD40-targeted *Ad5-luc1* or with the CD40-targeted *Ad5-huPSMA.* This immunization was repeated at 14 days; at 24 days from initiation of the immunization regimen, the mice were sacrificed and the spleens were removed and T-cells cultured. T-cells from the mice were re-stimulated for 6 days with γ-irradiated 293 cells infected with *Ad5-huPSMA*. Following re-stimulation, a ^51^Cr release CTL assay was performed with the stimulated CTL as effector cells (E) and RM-1 cells targets (T) at the indicated E:T ratios. Cytotoxicity was measured using parental RM-1 target cells, the RM-1 clonal cell lines 1 and 3 expressing PSMA, or the RM-1 clonal cell lines 2 expressing GFP. IFNγ induces the expression of several components of the antigen-processing machinery, leading to enhanced presentation of peptides in the context of HLA class I molecules on the cell surface [25]. Therefore, we decided to enhance CTL lysis of target cells by infecting them with an Ad vector expressing the murine interferon-gamma (*Ad5-IFNγ*).

T-cells from mice in the vaccination group receiving DCs infected with the CD40-targeted *Ad5-luc1* did not acquire CTL activity *(* *Fig. 7A* *).* Likewise, there was no specific lysis using target cells treated with *Ad5-IFNγ (* *Fig. 7B* *).* However, as shown in *Fig. 7C,* T-cells from mice in the vaccination group receiving DCs infected with the CD40-targeted *Ad5-huPSMA* exhibited weak specific lysis against RM-1-PSMA clone 3 cells (with a significance of P<0.05 at E:T ratios of 100∶1, 50∶1 and 25∶1) and RM-1-PSMA clone 1 cells (with a significance of P<0.05 at E:T ratios of 100∶1 and 50∶1), compared to control RM-1 cells or to RM-1-GFP clone 2 cells. Importantly, as shown in *Fig. 7D*, CTL lytic activity was significantly enhanced in RM-1-PSMA clone 1 and RM-1-PSMA clone 3 cells, which were pre-treated for 24 h with *Ad5-IFNγ.* However, neither RM-1 cells nor RM-1-GFP clone 2 cells, which were also pre-treated with *Ad5-IFNγ* showed any specific lysis. The differences between control RM-1 cells and either of the two cell lines expressing PSMA (RM-1-PSMA clone 1 or RM-1-PSMA clone 3) were statistically significant at all E:T ratios (P<0.01).

### CTL Assay after Vaccination with *Ad5-huPSMA*

The following experiment tested the functionality of the CD40-targeted Ad5-huPSMA to deliver an antigen to DCs in situ. In this experiment, DC-mediated CTL activation was assessed after direct intraperitoneal (i.p.) injection of the CD40-targeted Ad5-huPSMA. As shown in the vaccination schema in Fig. 8, C57BL6 mice were immunized i.p. with 1×10^8^ ifu of either the CD40-targeted Ad5-luc1 or the CD40-targeted Ad5-huPSMA. This immunization was repeated at 14 days with 1×10^8^ ifu of the CD40-targeted Ad. At 24 days from initiation of the immunization regimen, the mice were sacrificed and the spleens were removed and T-cells cultured. T-cells from the mice were re-stimulated as described above, followed by a classic ^51^Cr release CTL assay. Cytotoxicity was measured by ^51^Cr release from RM-1 target cells or RM-1 clonal cell lines expressing either PSMA or GFP.

As shown in Fig. 8A, T-cells from naive mice exhibited no specific lysis against any of the RM-1 cell lines. In, addition, no specific lysis was detected when the target cells were pre-treated with Ad5-IFNγ (Fig. 8B). Similarly, when mice were vaccinated in situ with the CD40-targeted Ad5-luc1, no specific lysis against any of the RM-1 cell lines was observed, regardless of their pre-treatment with Ad5-IFNγ (Fig. 8C and Fig. 8D). When mice were vaccinated with the CD40-targeted Ad5-huPSMA, RM-1-PSMA clone 3 did show weak CTL lysis. However, no significant ^51^Cr release was observed neither with parental RM-1 cells, nor RM-1-GFP clone 2 or RM-1-PSMA clone 1 (Fig. 8E). Significantly, as shown in Fig. 8F, specific lysis was dramatically enhanced in RM-1-PSMA clone 1 and RM-1-PSMA clone 3, when those cells were pre-treated with Ad5-IFNγ. Importantly, neither RM-1 parental cells nor RM-1-GFP clone 2, which were also pre-treated with Ad5-IFNγ showed any specific lysis.

### Assay of RM-1 Cells to Natural Killer (NK) Cell-mediated Cytotoxicity

NK cells play a major role in host defense against developing tumors and virally infected cells; thus, we investigated the sensitivity of RM-1 cells to NK cell-mediated cytotoxicity. To determine the sensitivity of the RM-1 and RM-1-PSMA clone 3 cell lines as targets for NK cell lysis, a chromium release assay was performed using mouse splenocytes. Although NK cells were not purified, they represent the only cell population capable of lysing the effector cells in this assay. As a control, an NK-sensitive cell line (YAC-1 mouse lymphoma cells) was also assayed. As shown in *Fig. 9A*, both the RM-1 and RM-1-PSMA clone 3 cell lines were insensitive to lysis by NK cells compared with YAC-1 cells. In the 4-hour incubation, very little or no cytolytic activity was detected, even in RM-1 and RM-1-PSMA clone 3 cells pretreated with *Ad5-IFNγ*. These results suggest that RM-1 cells are resistant to NK-mediated killing. To gain insight into the mechanism responsible for NK resistance, expression of NKG2D ligands on RM-1 clone 3 cells was investigated, since the expression of NKG2D ligands has been previously demonstrated to play important role in NK-mediated killing. As shown in *Fig. 9B*, the mean fluorescence intensities of immunostaining the murine NKG2D ligands H60, Rae-1, and MULT-1 were lower in RM-1 cells compared to YAC-1 cells (an NK-sensitive cell line) used as positive control. Similar low expression of NKG2D ligands was observed in RM-1 cells pretreated with *Ad5-IFNγ* (data not shown). These results suggest that low expression of NKG2D ligands in RM-1 cells may be involved in the resistance to NK-mediated killing we observed.

### Tumor Response after Vaccination *in vivo*

Finally, we asked if CD40-targeted *Ad5-huPSMA* vaccine could induce an immune response to control tumor growth. In this experiment, C57BL/6 mice were immunized by intraperitoneal injection of 1×10^8^ ifu of untargeted *Ad5-huPSMA* or CD40-targeted *Ad5-huPSMA*. The animals received a boost immunization at 10 days after the initial immunization. At the 14th day after the second immunization, the mice were challenged subcutaneously with 4×10^5^ RM-1 parental cells or RM-1-PSMA clone 1 cells. Three days later, the treatment groups were injected at the site of tumor cell injection with 1×10^8^ ifu of *Ad5-IFNγ* or with normal saline. As shown in *Fig. 10*, immunization with CD40-targeted *Ad5-huPSMA* alone or with non-targeted *Ad5-huPSMA* + *Ad5-IFNγ* similarly diminish tumor growth in animals challenged with RM-1-PSMA cells. This tumor growth was significantly delayed compared with the animals immunized with the CD40-targeted *Ad5-huPSMA* but challenged with parental RM-1 cells (that do not express the human PSMA antigen). Importantly, mice immunized with the CD40-targeted *Ad5-huPSMA* and *Ad5-IFNγ* demonstrated the greatest inhibition of tumor growth when challenged with RM-1-PSMA cells compared with the other groups.

## Discussion

In this study, we evaluated a prostate cancer vaccine based on adenoviral vector delivering PSMA antigen targeted by means of a bispecific adapter approach to CD40 expressing DCs. PSMA is a prostate tumor antigen that is widely expressed on the surface of prostate cancer cells. Generation of an appropriate immune response against tumor-specific antigens leading to eradication of tumors is of prime importance. Thus, our main goal in the study was to determine a proof-of-principle in using the Ad vaccine to directly stimulate DCs *in vivo* to target PSMA expressing prostate cancer cells. The major hurdle in targeting dendritic cells by means of adenoviral vectors is the lack of the native Coxsackie and Adenovirus receptor on the cell surface. We demonstrated expression of PSMA upon infection of DCs using a CD40-targeted *Ad5-huPSMA* vector, while using an untargeted vector resulted in undetectable PSMA expression. A major advantage using the adapter-based CD40-targeting approach is flexibility to use different sets of Ad vectors expressing unique tumor associated antigens. Our results also have relevance that would encourage the use of Ad vectors in immunotherapies targeting additional cancers in addition to prostate cancer.

MHC molecules play a critical role in the immune response against target antigens. Several studies have reported that MHC class I allele is frequently down regulated in as many as 85% of prostate tumors. The loss of MHC expression may be one of the potential mechanisms by which cancer cells evade host immunosurveillance. We show in this study that the parental RM-1 cells and their derivative clonal cell lines do not express detectable levels of MHC class I on their cell surface. However, pretreatment of RM-1 cells with *Ad5-IFNγ* led to significant up regulation of MHC class I as evident by flow cytometry analysis. Furthermore, since TAP1 and TAP2 form important components of the antigen presentation pathway, we studied their expression in RM-1 cells by Western blot analysis. These results indicate that RM-1 cells express low levels of TAP1 and TAP2, which may explain their propensity to form tumors and evade the immune response. Importantly, infection with *Ad5-IFNγ* induces TAP1 and TAP2 expression in the RM-1 cell lines. Thus, these results suggest that effective vaccine therapy in the context of the RM-1 model requires combination vaccine therapy using an immunomodulatory molecule to stimulate antigen presentation in target cells.

DCs play a functional role in eliciting cellular immune response against tumor specific antigens. We assessed the ability of CD40-targeted Ad infection on DC-mediated CTL activation in an *in vitro* ^51^Cr release CTL assay. We found efficient killing of the target RM-1 clone 1 and clone 3 cells expressing PSMA antigen by the T cells obtained from mice injected with DCs infected with *Ad5-huPSMA*. This lytic activity was further enhanced by infection of cells with an Ad vector expressing murine IFNγ. However, the CTL killing activity was absent in the group of animals receiving DCs infected with untargeted vector *Ad5-luc1*. The efficacy of CD40-targeted *Ad5-huPSMA* to deliver the antigen to DCs and induce CTL response was further tested using an *in vivo* CTL assay. Our data demonstrate weak killing of target RM-1-PSMA clone 3 by the T cells isolated from the mice immunized with *Ad5-huPSMA* as opposed to the animals treated with *Ad5-luc1*. This CTL lytic activity was dramatically enhanced in the PSMA expressing RM-1 clonal cell lines upon the treatment with *Ad5-IFNγ*, emphasizing on the significance of the combination vaccine therapy using immunomodulatory molecules to stimulate the CTL activity against tumor antigens.

In our experiments, we observed the most significant delay in the growth of RM-1-PSMA tumors in mice immunized with the CD40-targeted *Ad5-huPSMA* when the mice were also administered *Ad5-IFNγ*. Mice challenged with RM-1 cells that do not express human PSMA did not show inhibition of tumor growth. This suggests that the DC-based vaccine may confer resistance to the prostate tumor growth *in vivo* in an antigen specific manner. However, mice immunized with CD40-targeted *Ad5-huPSMA* without *Ad5-IFNγ*, and challenged with RM-1-PSMA cells offered intermediate protection against tumors. Likewise, mice immunized with untargeted *Ad5-huPSMA* but including *Ad5-IFNγ*, and challenged with RM-1-PSMA cells offered similar intermediate protection against tumors. It is possible that weak CTL activity was sufficient to offer some protection in these contexts. It is also possible that IFN*γ* itself offered some indirect anti-cancer protection. IFN*γ* has been shown to be capable of bestowing increased sensitivity to Fas-mediated cell death in prostate cancer cells [26].

Although delayed, growth of RM-1-PSMA tumors ultimately resumed in mice immunized with the CD40-targeted *Ad5-huPSMA* co-treated with *Ad5-IFNγ*. Nonetheless, these mice demonstrated a prolonged survival advantage. In conclusion, our study may aid the development of dendritic cell based cancer gene therapy approaches for the therapeutic intervention of prostate carcinoma. Future studies are warranted to explore the mechanisms involved in mediating protection against the PSMA expressing tumors as well as to maximize the therapeutic effect.
